# Quality of Life Among Caregivers of Children with Special Needs in Kelantan, Malaysia: The Importance of Psychosocial Mediators

**DOI:** 10.21315/mjms2021.28.2.12

**Published:** 2021-04-21

**Authors:** Siti Nor Ismalina Isa, Ismarulyusda Ishak, Azriani Ab Rahman, Nur Zakiah Mohd Saat, Normah Che Din, Syarif Husin Lubis, Muhammad Faiz Mohd Ismail, Nur Riza Mohd Suradi

**Affiliations:** 1 Department of Basic Sciences, Faculty of Health Sciences, Universiti Teknologi MARA, Puncak Alam Campus, Selangor, Malaysia; 2 Biomedical Science Programme, School of Diagnostic & Applied Health Sciences, Faculty of Health Sciences, Universiti Kebangsaan Malaysia, Kuala Lumpur, Malaysia; 3 Department of Community Medicine, School of Medical Sciences, Universiti Sains Malaysia, Kubang Kerian, Kelantan, Malaysia; 4 Health Psychology Programme, School of Healthcare Sciences, Faculty of Health Sciences, Universiti Kebangsaan Malaysia, Kuala Lumpur, Malaysia; 5 School of Mathematical Sciences, Faculty of Science and Technology, Universiti Kebangsaan Malaysia, Bangi, Selangor, Malaysia

**Keywords:** caregivers, children with special needs, psychosocial, quality of life, stress

## Abstract

**Background:**

Quality of life (QoL) is an important aspect of well-being for the caregivers of children with disability, making it a noteworthy outcome. Little is known about the challenges faced by the caregivers in Asian countries and its association to their QoL. The purpose of this study was to examine a model describing the relationship between sociodemographic and disability-related factors on caregivers’ QoL, mediated by the caregivers’ psychosocial factors such as perceived stress, coping skills, and social support.

**Methods:**

A cross-sectional study was conducted involving caregivers of children with special needs in Kelantan, a state of Peninsular Malaysia. A total of 383 caregivers completed questionnaires measuring sociodemographics, disability-related factors, psychosocial factors and QoL outcome. Structural equation modelling was performed to examine the relations of the variables in the conceptual model.

**Results:**

In the final model, childcare dependency, caregiver’s age, financial support, negative perception of stress and maladaptive coping skills were significant predictors of QoL (*R^2^* = 0.65). Caregiver’s age had a direct effect on QoL, while financial support had an indirect effect on QoL via negative perception of stress. Childcare dependency had direct and indirect effects on QoL via negative perception of stress. Higher perceived stress was associated with lower QoL directly and indirectly via maladaptive coping skills. The final model fitted the data well (root mean square error of approximation [RMSEA] = 0.046; CFI = 0.923; χ^2^/df = 1.798).

**Conclusion:**

Intervention strategies to improve the QoL of caregivers should target the family unit and take into account the factors of child’s disability, demographic and caregiver’s psychosocial status.

## Introduction

The term children with special needs (CWSN) refers to children with disabilities or ‘special children’. Nowadays, disability and developmental problems in children have become an important health issue with improvements in healthcare (1). Although a nurturing home environment can minimise the impact of impairment and improve a child’s independence, it should also be noted that providing the high-quality care required by children with long-term disabilities may impact the health and quality of life (QoL) of caregivers (2). QoL is defined as the individual’s perception of their position in life in the context of their culture and value systems in which they live, and in relation to their goals, expectations, standards, and concerns. It is a broad concept incorporating an individual’s physical health, psychological state, level of independence, social relationships, personal beliefs and relationship to salient features of the environment (3).

Over the past few years, there has been growing attention given to various social and health dimensions to understand the impacts of child disability on family life, rather than focusing solely on parental psychological health. Researchers have shown that caregivers of children with developmental disabilities are more likely to report chronic illnesses, limited activity, more somatic symptoms, lower levels of general health, and symptoms of depression and higher levels of stress than caregivers of children with typical development (4–5). Caregivers were also reported to experience changes in family relationships, marital relationships and job status (2, 6). Self-stigma and stigma from the community may also disrupt the social life of the caregivers and consequently negatively affect their QoL (7). As much of the available data about the impact of child disability on the parental QoL pertain to Western countries, little is known about the challenges faced by caregivers of CWSN in Asia and its association to their QoL. Moreover, the issue of caregiver burden remains as a hidden issue among family members and communities in Malaysia due to socio-cultural constraints and lack of concern from the respective parties (8). When compared to developed countries, the integration between health, education and social welfare services systems in Malaysia is still weak in helping to reduce the burden of caregivers as well as to improve the QoL of CWSN and their family (1). In addition, past local studies have shown that the most unmet needs identified among the parents of CWSN was the need for information (97.8%), followed by unmet needs in social support (93.8%), community service (90.3%) and financial support (82.7%) (8). Families who experienced high unmet needs will consequently experience impacts on their QoL (9–10).

Previous studies have shown that the influence of sociodemographic characteristics, medical and psychosocial factors on the health-related quality of life (HRQoL) of caregivers is a dynamic process. In addition to direct associations, various conceptual frameworks have been developed to prove that demographic, medical and social variables not only directly, but also indirectly affect the health and well-being of caregivers. Caregivers’ HRQoL was found to be either directly or indirectly related with child’s age, caregiver’s age, marital status and chronic illness (11), number of children in the family (12), gross income (13) and socioeconomic status (14). With respect to child functioning factors, the child’s care dependency, number of hours of sleep per night and wearing of diapers were significantly predictive of caregiver’s HRQoL (11, 15). Moreover, a recent study also revealed that psychosocial variables are more consistent and powerful factors to HRQoL domains, rather than sociodemographics or the child’s functioning (15). There is evidence that psychological well-being and HRQoL are mediated or moderated by a number of psychosocial variables such as parental stress, coping strategies and social support. Higher levels of parental stress, more use of maladaptive coping strategies and less social support were all related to lower QoL outcomes (16–18).

**Figure 1:**
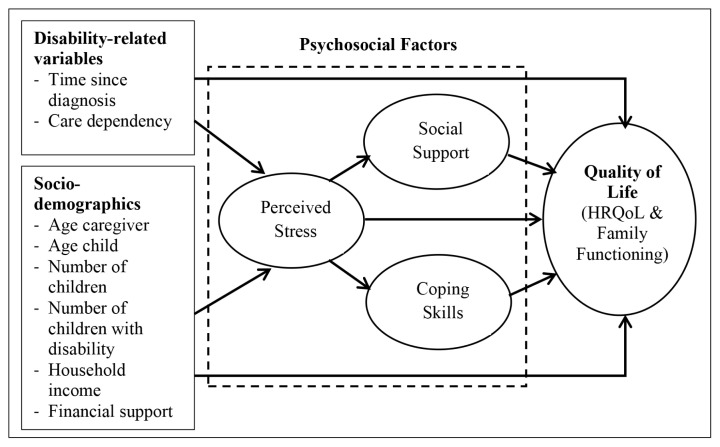
Conceptual model of relationships between sociodemographics, disability-related factors, psychosocial factors and QoL outcome in caregivers of CWSN

Some findings have explained the relationship between predictors of psychological health and QoL in the Malaysian population (19–21), but so far, most studies only explored direct predictors of psychological health and QoL by using traditional statistical analysis approaches such as multiple linear regression and binary logistic regression. Such approaches failed to determine the mediators and indirect pathways that occur between predictors and a caregiver’s QoL outcome. The aim of the present study was to develop a comprehensive model to determine the direct and indirect effects of sociodemographics, disability-related factors and psychosocial variables on QoL outcome of the caregivers of CWSN. In this study, the biopsychosocial model has been chosen as the theoretical framework because it emphasises individual dynamics and focuses on the interconnectedness of the individual’s biological, psychological, and environmental and social factors. Meanwhile, the proposed conceptual model is a modification of previous models, in which the factors of this model are appropriate to the population, the paths are not too complex and easier to interpret. Our conceptual model has adapted certain concepts and factors from the caregiving process model (22) and other HRQoL and family functioning models (11, 17). The selected concepts include background/context (sociodemographic factors), child characteristics (disability-related variables), intrapsychic factors (perceived stress), coping factors (coping skills and social support) and outcome (HRQoL and family functioning). The present study hypothesised that sociodemographic and child disability-related factors influenced QoL outcome directly and indirectly through perceived stress. This study also hypothesised that the relationship of perceived stress and QoL outcome was mediated by coping skills and social support. The hypothesised relationships based on the empirical findings and conceptual models from previous research are illustrated in Figure 1.

## Methods

### Participants and Setting

We conducted a cross-sectional study of community-based rehabilitation (CBR) centres and schools with special education integration programmes in Kelantan, the most northeastern state of Peninsular Malaysia. Malays are the major ethnic group, which comprises of 95.7% of Kelantan’s population, followed by Chinese (3.4%), Indians (0.3%) and others (0.6%). Kelantan has a gross domestic product (GDP) per capita of RM8,273 and a GDP growth of 4.1% in 2010. The state has the lowest level of urbanisation (42.4%) in Malaysia compared to other states (23).

The participants were parents or guardians (herein ‘caregivers’) of CWSN who registered with the Department of Social Welfare Malaysia and attending the CBR centres or schools with special education integration programmes. The inclusion criteria were as follows: i) those who were primary caregivers — mother or father or other family members who were responsible for caring of CWSN most of the time; ii) those who had a child with the diagnosis of Down syndrome, autism spectrum disorder (ASD), attention-deficit hyperactive disorder (ADHD), global developmental delays, intellectual disability, or specific learning disabilities; iii) their child aged of 18 years old and below and iv) their child stayed at home. Caregivers who were formal caregivers, absent during the study period, and/or demonstrated a severe mental illness were excluded from the study.

We employed a one-stage cluster random sampling method to select the study samples. The main advantage of this sampling method is that it saves cost and traveling time, as the centres are located far from one to another. A simple random sample of CBR centres and schools (clusters) was drawn from each region in the state of Kelantan. All caregivers in the selected CBR centres and schools meeting the criteria were then recruited into the study.

We explained the rationale of the study and obtained the respondents’ written consent before they were allowed to answer the questionnaire.

### Instruments

A set of structured questionnaires in the Malay version with five sections was used as the research instrument. The collected background information included both sociodemographic and disability-related variables. Sociodemographic variables included age, gender, race, monthly household income, and financial support received for the child and family per month. Disability-related variables included time since diagnosis (duration of disability), reported medical or health problems, types of diagnosis and care dependency. Care dependency was defined as the number of life domains on which their child needs care (eight items: physical, mobility, eating and drinking, medication use, coping with devices/tools, entertaining, contact with other children and education). These items were measured using a scale that ranges from 0 to 8, where 0 indicates that the child does not need support at all and score 8 indicates that the child needs full support (11).

The Perceived Stress Scale 10 items (PSS-10) by Cohen et al. (24) was used to measure the caregivers’ perceived stress. The questions in the PSS-10 ask about feelings and thoughts during the last month. PSS-10 has been validated by some local researchers who found that this scale is comparable to the original version for identifying stress in the Malaysian population (25). Exploratory factor analysis (EFA) showed that two factors have been extracted from this questionnaire. The first factor has six items (items 1, 2, 3, 6, 9 and 10) showing negative or stress perceptions, while four more items (items 4, 5, 7 and 8) show positive perceptions or control factors. A higher score on negative perception indicates higher perceived stress, while a higher score on the positive perception indicates lower perceived stress.

The Brief COPE inventory by Carver (26) was used to measure coping skills of the caregivers. Coping styles are classified into 14 subscales: i) self-distraction; ii) active coping; iii) denial; iv) substance use; v) use of emotional support; vi) use of instrumental support; vii) behavioural disengagement; viii) venting; ix) positive reframing; x) planning; xi) humour; xii) acceptance; xiii) religion and xiv) self-blame. The Brief COPE scale was translated from the original version in English into the Malay language and showed fairly good reliability and validity (27). Based on the evidence that these coping factors tend to be either be generally adaptive or problematic (28), some previous studies have used EFA to group subscales into two coping strategies, namely adaptive versus maladaptive coping (29). Adaptive strategies include the subscales of active coping, planning, positive reframing, acceptance, religion, humour, use of emotional support and use of instrumental support. Maladaptive coping includes subscales of self-distraction, denial, substance use, behavioural disengagement, venting and self-blame (28).

Social support of the caregivers was assessed using the MOS Social Support Survey Scale which consists of two main sections covering structural and functional support (30). The first part (item 1) is a single item structural indicator of social support. This question measures the number of close friends or relatives available to the participants. The second part contains 19 items (items 2 to 20) assessing four dimensions of functional social support that are emotional/informational support, instrumental support, affectionate support and positive social interaction.

QoL outcome of caregivers in this study was measured using the PedsQL™ Family Impact Module (31). PedsQL™ Family Impact Module is a parent-reported method which measures the impact of paediatric chronic medical conditions on the parental HRQoL and family functioning. This multidimensional instrument consists of eight subscales: i) physical functioning; ii) emotional functioning; iii) social functioning; iv) cognitive functioning; v) communication; vi) worry; vii) daily activities and viii) family relationships. The 36 items were rated on a 5-point Likert scale (0 = never a problem; 4 = always a problem). Items were reverse-scored and linearly transformed to a 0–100 scale (0 = 100; 1 = 75; 2 = 50; 3 = 25; 4 = 0) such that higher scores indicate better functioning (less negative impact). The original version of PedsQL™ Family Impact Module was translated into the Malay language and showed that the internal consistency reliability of all domains was above Cronbach’s alpha of 0.7 (32).

### Statistical Analysis

Descriptive statistics were calculated for all variables in the dataset. Correlation analyses were used to explore the strength of the relationship between the study variables. Structural equation modelling (SEM) using the analysis of moment structures (AMOS) version 21.0 software was performed to test hypotheses outlined in the conceptual model and to test whether the conceptual model fitted the data. This analytic approach involved a 2-step process. In the first step, confirmatory factor analysis (CFA) was used to test the validity and reliability of the measurement model of latent constructs. The second step focused on testing hypotheses about relationships among the variables in the structural model using path analysis. A parsimonious model is preferred in path analysis (33); therefore, only the significant correlations of the variables were taken into consideration when the initial hypothesised path model was developed. Several fit indices were considered to determine the goodness-of-fit of the measurement and structural model. The statistics included the root mean square error of approximation (RMSEA), with a desired value of less than 0.08, the comparative fit index (CFI) with desired values of greater than 0.90 and the χ^2^/df with a desired value of less than 3.0 (34). The final evaluation of the hypothesised model was made by examining the fitness criteria, and the model was re-specified and re-modified to obtain acceptable model fit. After obtaining the final structural model, the significance (*P*-value) of the direct, indirect and total effects of all the factors on QoL were calculated using a bootstrapping procedure in the modelling analysis. We used an alpha of 0.05 for the significance level.

## Results

### Characteristics of Participants

Demographics and disability-related variables are presented in Table 1. A total of 405 caregivers of CWSN were asked to participate in the study, of which 383 (94.6%) completed the questionnaires and were available for analysis. The mean age of the caregivers was 45.6 (SD = 9.40) years old and ranged from 18 to 75 years old. Almost all of the participants were the children’s biological parents (93%), of which 77% were female. Their median monthly household income was Ringgit Malaysia (RM)800, with minimum and maximum incomes of RM0 and RM11,000, respectively (USD1 = RM4.08). In terms of child diagnosis, most of the children were children with intellectual disability (36.8%) and children with Down syndrome (35.8%).

### Correlation Between Variables

The strongest correlation was shown between negative perception of stress and QoL outcome (*r* = −0.642, *P* < 0.001). The higher the level of negative perception of stress, the lower the level of HRQoL and family functioning of caregivers. A moderate negative correlation was shown between maladaptive coping and QoL (*r* = −0.439, *P* < 0.001). Higher use of maladaptive coping had a correlation with lower levels of HRQoL and family functioning. A moderate correlation was also shown between negative perception of stress and maladaptive coping (*r* = 0.411, *P* < 0.001), which means that the higher the level of negative perception of stress, the higher the use of maladaptive coping. Correlations between the study variables are presented in Table 2.

### Effects of Sociodemographics, Disability-Related Factors and Psychosocial Factors on QoL

**Figure 2:**
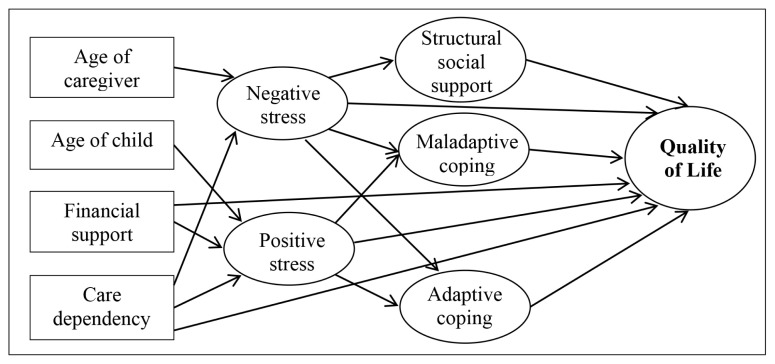
Initial hypothesised structural model with 17 pathways. Model fit indices RMSEA = 0.052; CFI = 0.896; *χ*^2^/df=2.039

**Figure 3:**
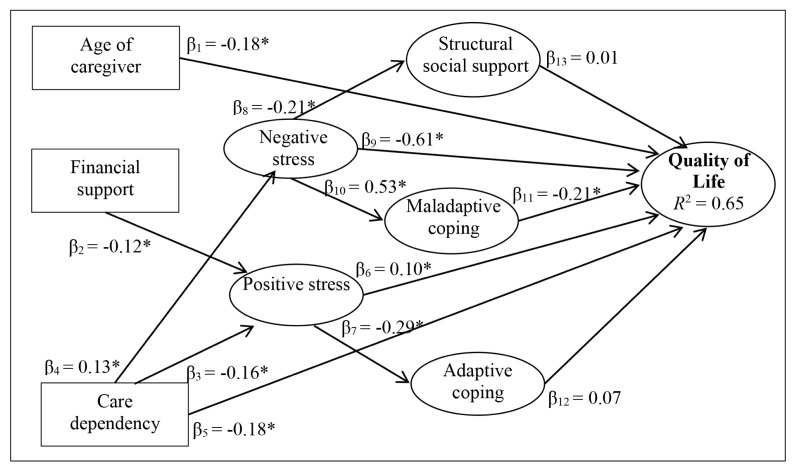
Final structural model of factors predicting QoL of caregivers of CWSN. Model fit indices RMSEA = 0.046; CFI = 0.923; *χ*^2^/df=1.798. * *P* < 0.05

Based on the literature review and results from the correlation analysis in this study, the initial hypothesised model was developed as illustrated in Figure 2. The initial hypothesised structural model did not result in a good fit to the data (RMSEA = 0.052; CFI = 0.896; χ^2^/df = 2.039). After considering the results of the initial hypothesised model and theoretical issues, some modifications were made. Figure 3 illustrates the final structural model that achieved acceptable fit indices (RMSEA = 0.046; CFI = 0.923; χ^2^/df = 1.798). Several significant direct and indirect effects of demographic, disability-related and psychosocial variables on QoL were found. In the final path model, child’s care dependency, caregiver’s age, financial support, negative perception of stress, and maladaptive coping skills showed significant total effects on QoL. The direct, indirect, and total effects of factors on QoL were calculated using the path analysis. For example, the impact of negative perception of stress on QoL involves 1 direct path (*β*_9_) and 1 indirect path (*β*_10_ × *β*_11_). The total effect (*β*_T_) was estimated by summating the direct effect and the indirect effects [(*β*_9_ + (*β*_10_ × *β*_11_)] (Figure 3).

Negative perception of stress was the strongest predictor of QoL of the caregivers (β_T_ = −0.72). An increase in the negative perception of stress (higher perceived stress) was associated with an increase in the use of maladaptive coping and a decrease in QoL directly and indirectly through maladaptive coping. Increased use of maladaptive coping was associated with decrease in QoL (*β*_T_ = −0.21). Care dependency had a significant direct effect on QoL as well as indirect effect on QoL via negative and positive perception of stress (*β*_T_ = −0.29). Higher levels of childcare dependency were associated with lower QoL among caregivers directly and indirectly via negative and positive perception of stress. Sociodemographic variables such as age of caregiver and financial support also were significantly affecting QoL of the caregivers. Age of caregiver had only a significant direct effect on QoL (*β*_T_ = −0.18), as older age was directly associated with poorer QoL. Financial support did not directly affect QoL. Higher reported financial support was associated indirectly with lower QoL via positive perception of stress (higher perceived stress) (*β*_T_ = −0.01). Additionally, the final model showed that structural social support, positive perception of stress, and adaptive coping had non-significant total effects on QoL. The direct, indirect, and total effects of the sociodemographic, disabilityrelated and psychosocial factors are shown in Table 3.

## Discussion

In this study, a single comprehensive model for determining the direct and indirect relationships between sociodemographics, child disability-related and psychosocial factors with caregivers’ QoL has been tested using structural equation modelling. The framework of the initial hypothesised model was based on a conceptual model derived from Western studies (11, 16–17, 22). The final model fitted the data well but appeared to be slightly different from our conceptual model. In the final model, childcare dependency, age of caregiver, financial support, negative perception of stress and maladaptive coping had significant direct and/or indirect relationships with the QoL of caregivers.

Within the disability-related factor, care dependency was the most important predictor with the QoL of caregivers. It influenced QoL both directly and indirectly through perceived stress. An increase in the level of children’s dependency on caregivers for care, leads to a lower QoL of their caregivers, directly and indirectly through its effect on caregivers’ stress. High childcare dependency was found to have increased the stress level of caregivers which in turn declined their QoL outcome. Qualitative findings have explained our finding that long-term dependence of the child on the caregivers can cause emotional stress, fatigue, sleep disorders and limited time which affect the QoL of caregivers (2, 35).

The current study has also revealed issues that have rarely been discussed in past studies. Two sociodemographic factors, specifically the age of caregiver and financial support, were found to have relationship with the caregiver’s QoL directly or indirectly. Age of caregiver had a direct negative effect on QoL, indicating older caregivers had lower levels of QoL. This finding is in accordance with a study in South Korea which similarly found older caregivers reported lower life satisfaction. The growing needs of children with disabilities and the worsening physical health of the caregivers as their age increases, coupled with fatigue due to long periods of care, ultimately caused a decline in life satisfaction amongst the caregivers (36). Interestingly, our study found that the financial support received by family caregivers had an indirect negative effect on QoL through perceived stress. In general, every child with special needs in Malaysia who attends the special education programme in schools or the CBR programme, receives a monthly allowance of RM150 per month from the government. However, some caregivers who reported receiving higher financial assistance were those who have more than one disabled child, were single mothers or from poor families who also received other financial assistance provisions from the Department of Social Welfare or other welfare agencies. Our findings implied that although this group of caregivers received various welfare assistance, they still felt it was insufficient to accommodate the lives of their families, and thus felt greater stress. Inadequate public financial assistance is a common issue affecting the QoL of the families of children with disabilities (37).

Furthermore, concerning caregivers’ psychosocial factors, perceived stress was found to be the key mechanism of the caregivers’ QoL outcome. Caregivers’ perceived stress affected QoL outcome both directly and indirectly through maladaptive coping skills. It also mediated the relationship of childcare dependency and financial support and QoL outcome. Nevertheless, the current study did not find significant relationship between social supports and QoL. The current study suggests that the higher the stress felt by the caregivers due to childcare dependency and financial pressure, the more they use adaptive and maladaptive coping strategies. However, the final model showed that only caregivers who use maladaptive coping had significant effect on declining of QoL outcome. Previous research found that maternal use of avoidant coping mechanisms such as distraction and disengagement was associated with increased levels of maternal depression and anger and also lower level of maternal well-being (38). On the other hand, use of cognitive/positive reframing (adaptive coping) was related with higher levels of maternal well-being and increased positive parental experience associated with raising a child with disability (38–39). The results of our study implied that the caregivers who had lower QoL were those who could not manage to adapt with the stress successfully. Hence, the development of supports that focus specifically on the development of positive coping skills is crucial to these caregivers in order to help them deal with the stress, thereby enhancing their QoL.

Several limitations of this study should be noted. Firstly, although the present study has included a considerable number of caregivers, the caregivers were drawn from only one state in Malaysia and belonged to the Malay ethnicity. Therefore, our results are not representative of the entire group of caregivers of CWSN in Malaysia who consists of different ethnic groups. Secondly, cross-sectional design was used in this study which does not allow inferences about causality. Therefore, relationships between QoL outcome and other variables should be interpreted with caution, and causality cannot be assumed. Thirdly, it is also important to note that assessment of the current study was based upon caregivers’ self-report and some of illiterate caregivers were interviewed, thus the results might be biased by individual response styles. Caregivers also were more likely to deny their stress and minimise their problems with HRQoL and family functioning, which might lead to underestimation of the negative impact of their child’s disability on them. Expectations of those illiterate caregivers who were interviewed might be lower, rather than them willingly reporting the problems, which could underestimate the impact of the disability. Notwithstanding these limitations, the results of the present study give more insight into the dynamics of caregivers’ QoL, especially in the context of the Asian population.

## Conclusion

This study highlights the importance of biopsychosocial factors on the HRQoL and family functioning of caregivers. The final structural model revealed that older caregivers were directly associated with lower QoL. Caregivers who received greater financial support were associated with lower QoL indirectly via caregiver’s perceived stress. Childcare dependency also had both direct and indirect negative effects on QoL via caregivers’ perceived stress. Meanwhile, perceived stress (negative perception) had direct and indirect negative effects on QoL via maladaptive coping.

The final model produced by this study can be tested and confirmed in future studies by involving larger sample sizes and covering every state in Malaysia. It is recommended that future work would benefit from access to a larger and more varied pool of participants to enhance its generalisability. In practice, interventions using family-centred models should be emphasised and extensively implemented by healthcare, education and other service providers who deal with CWSN. Greater emphasis should be placed on caregivers who have higher risk of stress, such as caregivers of children with more severe disabilities, older caregivers, and those coming from poor families.

## Figures and Tables

**Table 1:** Characteristics of caregivers and their CWSN (*n* = 383)

Characteristics	Mean (SD)	*n* (%)
Sociodemographic variables		
Age caregiver (years)	45.6 (9.40)	
Relationship to the child		
Father/Mother		356 (93.0)
Grandfather/Grandmother		5 (1.3)
Siblings		11 (2.9)
Others		11 (2.9)
Gender (female)		295 (77.0)
Marital status		
Married		344 (89.8)
Not married		39 (10.2)
Number children per family		
One to five		234 (61.1)
> Five		149 (38.9)
Number children with disability		
One		337 (88.0)
≥ Two		46 (12.0)
Monthly household income (RM)	800.0 (1000.00)^a^	
≤ 2000		323 (84.3)
> 2000		60 (15.7)
Financial support (RM)	150.0 (0.00)^a^	361 (94.3)
Age child (years)	11.7 (4.29)	
Gender child (boys)		238 (62.1)
Disability-related variables		
Time since diagnosis (years)	7.9 (4.83)	
Types of diagnosis		
Down syndrome		137 (35.8)
ADHD		17 (4.4)
Autism/ASD		35 (9.1)
Global developmental delay		30 (7.8)
Intellectual disability		141 (36.8)
Specific learning disabilities		23 (6.0)
Care dependency*	14.0 (23.00)^a^	

Notes:

a median (IQR);

* scale 0–8 (high score representing high dependency)

**Table 2:** Correlations of study variables (*n* = 383)

Variables	Perceived stress — negative perception	Perceived stress — positive perception	Adaptive coping	Maladaptive coping	Structural social support	Functional social support	QoL (HRQoL & family functioning)
1. Age child	−0.052	0.101*					0.016
2. Age caregiver	−0.141**	0.075					−0.067
3. Number children with disability	0.064	−0.021					−0.073
4. Number of children	−0.046	−0.048					−0.015
5. Monthly household income	−0.089	0.077					0.097
6. Financial support	0.095	−0.113*					−0.127*
7. Care dependency	0.126*	−0.136*					−0.219**
8. Time since diagnosis	−0.084	−0.002					0.007
9. Perceived stress **—** negative perception	1	−0.361**	0.161**	0.411**	−0.187**	−0.073	−0.642**
10. Perceived stress **—** positive perception		1	−0.239**	−0.103*	0.079	0.008	0.302**
11. Adaptive coping			1				−0.129*
12. Maladaptive coping				1			−0.439**
13. Structural social support					1		0.161**
14. Functional social support						1	0.026
15. QoL (HRQoL & family functioning)							1

Notes:

* *P* < 0.05;

** *P* < 0.01;

The bold correlation coefficients (*r*) are significant correlations that used to develop paths between variables in initial structural model

**Table 3:** Direct effect, indirect effect and total effect (standardised regression coefficients) of all factors in the model finalstructural

Path relationships	Direct effect (*β*)	Indirect effect ( *β*)	Total effect (*β*_T_)
Financial support → Positive stress	−0.12*		−0.12*
Financial support → Adaptive coping		0.03*	0.03*
Financial support → QoL		−0.01*	−0.01*
Care dependency → Negative stress	0.13*		0.13*
Care dependency → Positive stress	−0.16*		−0.16*
Care dependency → Structural social support		−0.03*	−0.03*
Care dependency → Adaptive coping		0.04*	0.04*
Care dependency → Maladaptive coping		0.07*	0.07*
Care dependency → QoL	−0.18*	−0.11*	−0.29*
Age of caregiver → QoL	−0.18*		−0.18*
Negative stress → Maladaptive coping	0.53*		0.53*
Negative stress → Structural social support	−0.21*		−0.21*
Negative stress → QoL	−0.61*	−0.11*	−0.72*
Positive stress → Adaptive coping	−0.29*		−0.29*
Positive stress → QoL	0.10*	−0.02	0.09
Adaptive coping → QoL	0.07		0.07
Maladaptive coping → QoL	−0.21*		−0.21*
Structural social support → QoL	0.01		0.01

Notes:

* *P* < 0.05;

The bold path relationships are the significant total effects of the variables on QoL for the final structural model
